# [Corrigendum] LSKL peptide alleviates subarachnoid fibrosis and hydrocephalus by inhibiting TSP1-mediated TGF-β1 signaling activity following subarachnoid hemorrhage in rats

**DOI:** 10.3892/etm.2025.12953

**Published:** 2025-08-19

**Authors:** Fan Liao, Gaofeng Li, Wen Yuan, Yujie Chen, Yuchun Zuo, Kauthar Rashid, John H. Zhang, Hua Feng, Fei Liu

Exp Ther Med 12:2537-2543, 2016; DOI: 10.3892/etm.2016.3640

Subsequently to the publication of the above article, a cocnerned reader drew to the Editor’s attention that, for the western blot experiments shown in Figs. 2C and 3B, the β-actin control bands shown in these figure parts were strikingly similar in appearance, albeit they were shown in an inverted orientation in one of the figures relative to the other, and the western blots may have been performed under the same experimental conditions.

After consulting the authors about this apparent anomaly, they realised that Fig. 3 had inadvertently been assembled incorrectly; moreover, they were able to present the raw data for the western blots for all five experimental repeats relating to these figures. A revised version of Fig. 3, showing replacement data from one of the repeated experiments in Fig. 3B, is shown on the next page. Note that the error made in assembling the published version of Fig. 3 did not have a major impact on either the overall results or on the conclusions reported in this study. All the authors agree with the publication of this corrigendum, and are grateful to the Editor of *Experimental and Therapeutic Medicine* for granting them the opportunity to publish this; furthermore, they apologize to the readership for any inconvenience caused.

## Figures and Tables

**Figure 3:**
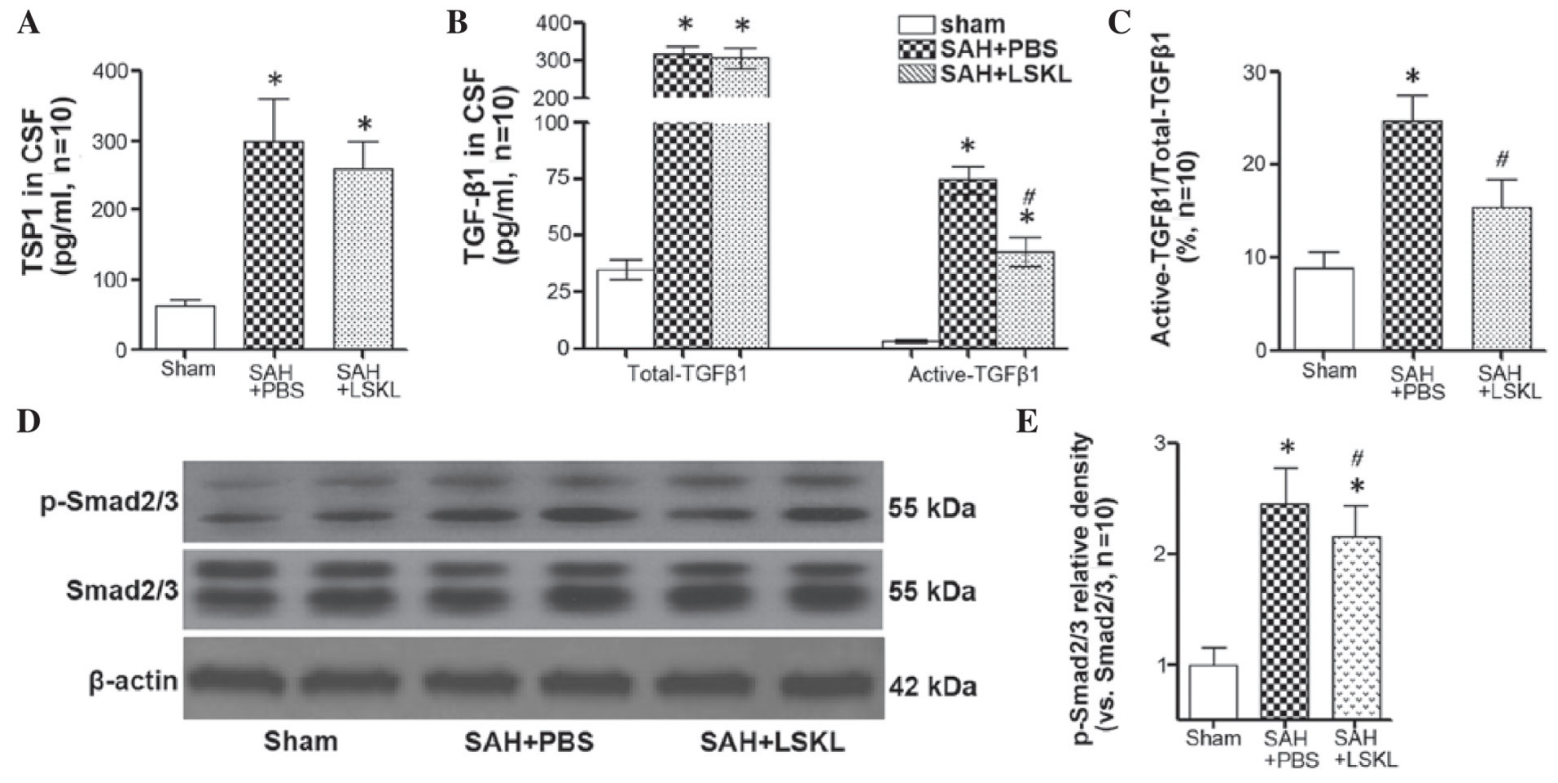
LSKL peptide inhibited TSP1-mediated TGF-β1 signaling activity following SAH. Quantitative analyses of (A) TSP1 and (B) total TGF-β1 and active TGF-β1 in the CSF on days 3–5 after SAH. (C) Ratio of active TGF-β11 to total TGF-β1 in the CSF on days 3–5 after SAH. (D) Representative western blot bands of p-Smad2/3 and (E) quantitative analyses of p-Smad2/3 expression on day 5 after SAH. Relative densities of each protein have been normalized against the sham group. Data are expressed as the mean ± standard error of the mean (n=10). ^*^P<0.05 vs. the sham group; ^#^P<0.05 vs. the SAH+PBS group. SAH, subarachnoid hemorrhage; CSF, cerebrospinal fluid; TSP1, thrombospondin-1; LSKL, leucine-serine-lysine-leucine; PBS, phosphate buffer solution.

